# High expression of TACC3 in esophageal squamous cell carcinoma correlates with poor prognosis

**DOI:** 10.18632/oncotarget.3190

**Published:** 2015-02-10

**Authors:** Zhi-Liang Huang, Zhi-Rui Lin, Yi-Ren Xiao, Xun Cao, Lin-Chun Zhu, Mu-Sheng Zeng, Qian Zhong, Zhe-Sheng Wen

**Affiliations:** ^1^ State Key Laboratory of Oncology in South China, Collaborative Innovation Center for Cancer Medicine, Sun Yat-Sen University Cancer Center, Guangzhou, Guangdong, China; ^2^ Department of Thoracic Oncology, Sun Yat-Sen University Cancer Center, Guangzhou, China; ^3^ South China Institute for Stem Cell Biology and Regenerative Medicine Key Laboratory of Regenerative Biology, Guangzhou Institutes of Biomedicine and Health, Chinese Academy of Sciences, Guangzhou, China; ^4^ Department of Critical Care Medicine, Sun Yat-Sen University Cancer Center, Guangzhou, China

**Keywords:** TACC3, Esophageal squamous carcinoma (ESCC), prognostic biomarker, immunohistochemistry

## Abstract

To analyze the expression of the transforming acidic coiled-coil protein 3 (TACC3) in esophageal squamous cell carcinoma (ESCC) samples, and to identify whether TACC3 can serve as a biomarker for the diagnosis and prognosis of ESCC, qPCR, western blotting and immunohistochemistry staining (IHC) were utilized to detect the expression of TACC3. Furthermore, cell growth, colony formation, migration ability and the epithelial-mesenchymal transition markers of ESCC cells in which TACC3 were knocked-down were measured. The mRNA and protein levels of TACC3 were higher in ESCC specimens compared to non-tumorous esophageal epithelial tissues. IHC results revealed TACC3 expression was significantly correlated to differentiation (*p* = 0.017) and lymphoid nodal status (*p* = 0.028). The patients with high-expression of TACC3 had a significantly poor prognosis compared to those of low-expression (*p* = 0.017), especially in the patients at stages I–II (*p* = 0.028). Multivariate analysis indicated that TACC3 expression was an independent prognostic factor for ESCC patients (*p* = 0.025). Knockdown of TACC3 inhibited the ability of cell proliferation, colony formation and migration. This study first identifies TACC3 not only as a useful biomarker for diagnose and prognosis of ESCC, but also as a potential therapeutic target for patients with ESCC.

## INTRODUCTION

Esophageal cancer, the eighth most common cancer in the world, is composed of two main histologic types: squamous cell carcinoma (ESCC) and adenocarcinoma (EAC) [1]. More than 480,000 patients are diagnosed with esophageal cancer and 400,000 of the patients die of esophageal cancer, annually [2]. More than 50% incidence and mortality happened in china. In North-Central China, referred to as the “esophageal cancer belt”, 90% of cases are squamous cell carcinomas [3]. Despite the efforts to improve the effectiveness of the combination of surgical approach and radiochemotherapy, there is an immense clinical need for new therapeutic strategies and molecular targets.

The allelic losses at chromosomes 3p, 5q, 9p, 9q, 13q, 17p, 17q and 18q, as well as mutations of p53 (missense), Rb (deletions), cyclin D1 (amplifications) and c-myc (amplifications) were commonly found in esophageal cancer [4]. However, the clear cellular and molecular mechanisms leading to ESCC have not been systematically evaluated to date. A lack of efficient molecular predictors and exact mechanisms for esophageal cancer are the critical barriers for developing clinical useful strategies for esophageal cancer managements.

The transforming acidic coiled-coil protein (TACC) family is characterized by a conserved C-terminal “TACC domain”, essential for the interaction with tubulin and microtubules and has been known to play a key role in the regulation of centrosome and microtubule dynamics [5–8]. There are three TACC proteins identified in human: TACC1, TACC2 and TACC3. TACC3, originally isolated from 4p16.3 region [9], is an Aurora A kinase target strongly concentrated at centrosomes throughout the cell cycle and identifies a member of centrosomal proteins family that can regulate microtubules formation [6, 10–12]. It is reported that TACC3 is a promising cancer chemotherapy target and knockdown of TACC3 may efficiently improve the chemosensitivity of cancer cells by modulating a premature senescence program [13, 14]. TACC3 also appear to play a basic role during early stages of differentiation in normal tissues [15]. TACC3 deficiency has been demonstrated to link with a high rate of p53-mediated apoptosis [16] and suppression of EMT phenotype through the activation of PI3K/Akt and ERK signaling pathways [17, 18]. Recently, TACC3 has been demonstrated as a tumor-associated gene involved in the development of cancer of multiple myeloma [9, 19], lung [20], bladder [21, 22], cervix uteri [18], breast [23], thyroid gland [24], and glioblastoma [25]. Nevertheless, the prognostic significance and function of TACC3 in human ESCC has not yet been well elucidated.

Therefore, this study was aimed to investigate the expression of TACC3 and explore the role of TACC3 in ESCC. In this study, quantitative reverse transcriptase PCR (qRT-PCR), Western blot analysis, and immunohistochemistry (IHC) methods were used to examine TACC3 mRNA and protein expression. Correlation of TACC3 expression with clinicopathological features specific to ESCCs was also assessed. In addition, functional studies were performed to identify knockdown of TACC3 could inhibit the proliferation, colony formation ability and epithelial mesenchymal transition (EMT) in ESCC cells. In all, our findings indicate that TACC3 was high expression and played an important role in ESCC. It may serve as an independent prognostic biomarker for ESCC patients, peculiarly those with early stages.

## RESULTS

### TACC3 is overexpressed in ESCC cell lines and fresh tissues

**Figure 1:**
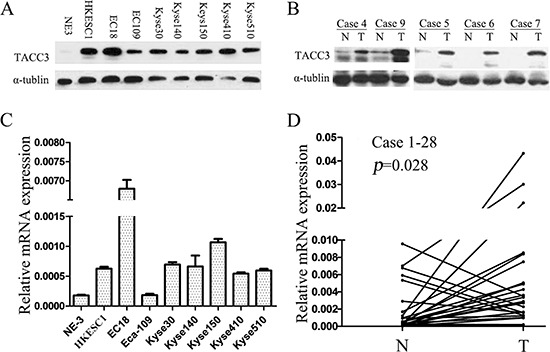
TACC3 expression is frequently upregulated in ESCC cell lines and esophageal tissue TACC3 protein and mRNA levels in a panel of ESCC cell lines including Eca-109, EC18, HKESC1, KYSE30, KYSE140, KYSE150, KYSE410, and KYSE510, compared as the immortalized normal human esophageal epithelial cell, NE3 **(A, C)** and in 28 pairs of matched ESCC and non-tumor tissues **(B, D)**. mRNA levels are presented as means ± SD and normalizing to the housekeeping gene β-actin in qRT-PCR. N, matched noncancerous tissue; T, tumor tissue; ESCC, esophageal squamous cell cancer.

To investigate the TACC3 expression in ESCC, qRT-PCR and western blotting were performed in both an immortalized normal human esophageal epithelial cell line, NE3, and a panel of ESCC cell lines including Eca-109, EC18, HKESC1, KYSE30, KYSE140, KYSE150, KYSE410, and KYSE510, respectively. TACC3 expression was barely detectable in NE3, whereas a notably higher level of TACC3 expression was showed in most of ESCC cell lines except Eca109, a well differentiated ESCC cell line (Figure 1A and 1C). Furthermore, we performed qRT-PCR and western blotting in 28 pairs of matched ESCC tissue and adjacent noncancerous tissue to examine whether the high expression of TACC3 occurred in ESCC patients. The protein expression of TACC3 in the tumor tissues was mostly higher than those paired adjacent normal samples (Figure 1B). Consistently, the expression level of TACC3 in ESCC tissues is significantly higher than the level in matched normal tissues by quantitative analysis (Figure 1D). In conclusion, TACC3 was frequently upregulated in both ESCC cell lines and tissues.

### Association between TACC3 expression and progression of ESCC

**Figure 2:**
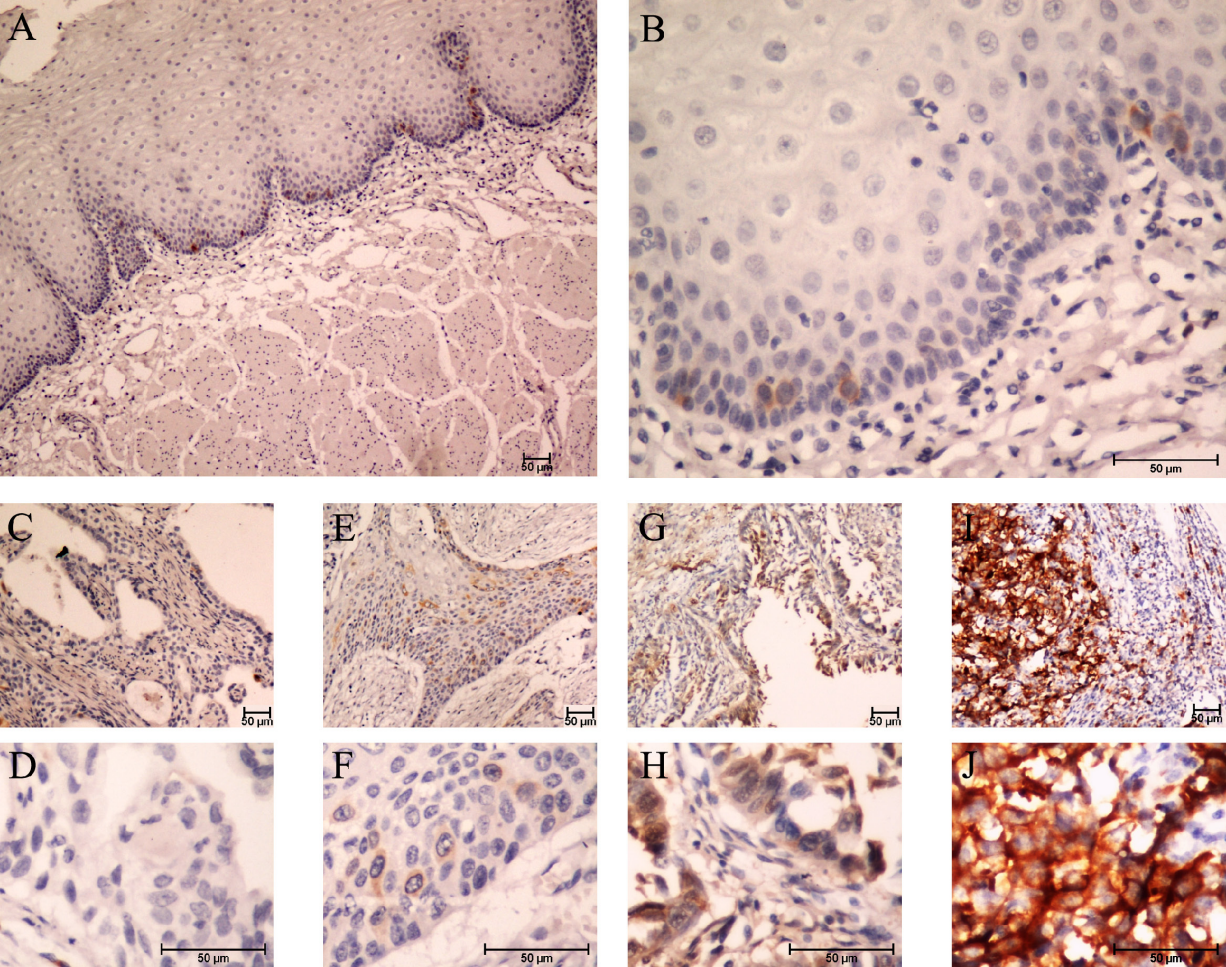
Expression of TACC3 in ESCC tissues by immunohistochemistry staining **(A** and **B)** negative TACC3 staining in normal esophageal epithelium tissue (negative control) (A) 100×, (B) 400×; **(C** and **D)** negative staining of TACC3 in ESCC tissue (C) 100×, (D) 400×; **(E** and **F)** weak staining of TACC3 in cytoplasm (E) 100×, (F) 400×; **(G** and **H)** moderate staining of TACC3 in cytoplasm (G) 100×, (H) 400×; **(I** and **J)** strong staining of TACC3 in cytoplasm (I) 100×, (J) 400×.

**Figure 3:**
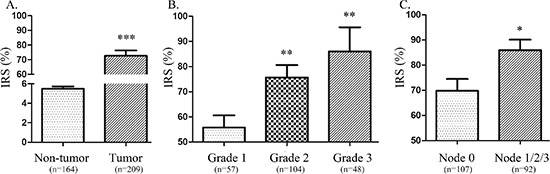
Expression level of TACC3 corresponded with the progression of ESCC **(A)** A distinct distinction between TACC3 expression in ESCC tissues and non-tumor esophageal epithelial tissues was detected. **(B)** The expression of TACC3 increased progressively from Grade 1 to Grade 3 of ESCC. **(C)** Moreover, TACC3 expression in cases with lymphoid nodal metastasis was significantly higher than the expression in patients without lymphoid nodal metastasis. Data presented as means ± SD; N, normal esophageal tissues; IRS, the immunoreactivity score (*p < 0.05, **p < 0.01, ***p < 0.001).

To further examine whether high expression of TACC3 protein is correlated with the clinical progression of ESCC, 209 ESCC and 164 matched adjacent esophageal epithelial tissues were subjected IHC staining with a human TACC3 antibody. Figure 2A to 2J were the representative results of IHC showing that TACC3 was localized to the cytoplasm. Tumors with an IRS exceeding 60% were deemed to be high expression of TACC3. Of the 209 tumors, 107 (51%) were with high expression of TACC3, whereas none of the 164 adjacent esophageal epithelial tissues presented high expression of TACC3. The IRS shows a striking difference of the expression of TACC3 in tumors and non-tumors tissues (Figure 3A). Interestingly, TACC3 expression in each tumor tissue was higher than that of the corresponding matched non-tumor tissue (Supplementary Table S1). Furthermore, the expression level of TACC3 was correlated with the differentiation of ESCC (Figure 3B). The average expression level of TACC3 increased progressively through Grade 1 to 3. Moreover, TACC3 expression in ESCC patients with lymphoid nodal metastasis was significantly higher than that with no lymphoid nodal metastasis (Figure 3C). Table 1 compares the demographic characteristics and tumor characteristics according to TACC3 expression. TACC3 expression was significantly associated with differentiation (Grade classification, Table 1, *p* = 0.017) and lymphoid nodal status (pN classification, Table 1, *p* = 0.028). However, there was no relationship between patient genders, age, smoking status, alcohol intake, pT status or stage. Our finding suggested that increased expression of TACC3 is associated to ESCC development and progression.

**Table 1:** Characteristics of the patients

characteristics	No of patients	Expression of TACC3		
		Low	High	*P*
patients	109	102	107	
Age				
Median	57			
Range	32–80			
≤ 57	106	61	45	
> 57	103	62	41	0.697
Gender				
Female	59	26	33	
Male	150	76	74	0.390
Smoking Status				
Smoker	130	66	64	
Non-smoker	79	36	43	0.466
Alcohol Intake				
Yes	51	27	24	
No	158	75	83	0.497
Grade				
Grade 1	57	37	20	
Grade 2	104	45	59	
Grade 3	48	20	28	0.017
pT Status				
pT 1	4	2	2	
pT 2	62	30	32	
pT 3	143	70	73	0.996
pN Status				
pN 0	117	65	52	
pN 1/2/3	92	37	55	0.028
Stage				
I–II	132	68	64	
III	77	34	43	0.305

*p* values were calculated by Pearson's Chi-square test.

### Correlation of TACC3 expression and overall survival

**Figure 4:**
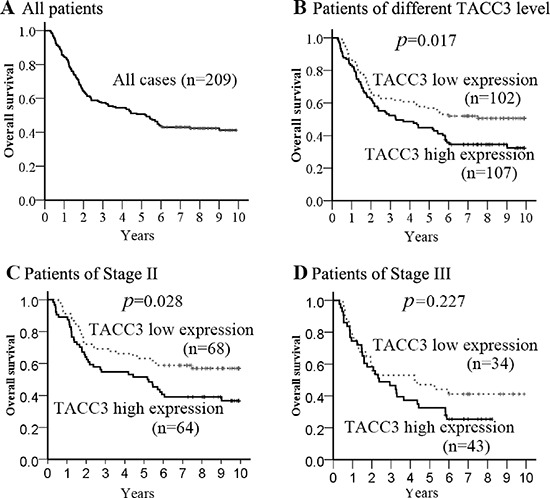
Kaplan–Meier estimates of the probability of survival **(A)** The five-year overall survival (OS) rate was 50.7% of 209 ESCC patient; **(B)** High TACC3 expression level was significantly correlated to OS (* p* = 0.017) in all ESCC patients. Furthermore, cases were stratified by pathological stage. **(C)** High TACC3 expression level was significantly associated with OS (*p* = 0.028) in ESCC patients at Stage II; **(D)** No significant difference in 5-year OS rate was found between TACC3 high-expression and low-expression in ESCC patients at Stage III.

Of the 209 patients in this study, the median follow-up period was 5.2 years (range, 0.3 to 10 years), with 121 cancer-related deaths at the final clinical follow-up. The 5-year overall survival rate was 50.7% for the total study population (Figure 4A). In the Kaplan–Meier analysis, OS was longer for patients with low TACC3 expression than those with high TACC3 expression (*p* = 0.017, median 6.0 vs. 3.7 years, Figure 4B). Further stratification of patient groups based on stage displayed that the correlation of low TACC3 expression and longer OS was statistically significant in Stage I–II patients with ESCC (*p* = 0.028, median 7.1 vs. 6.0 years, Figure 4C). However, in Stage III, there was no significant association between low TACC3 expression and longer OS (*p* = 0.227, median 1.9 vs. 1.6 years, Figure 4D).

Next, we examined OS using Cox proportional hazards model to determine whether TACC3 expression could serve as an independent predictor. A series of factors, including age, gender, smoking status, alcohol intake, grade, tumor location, surgery, pathological staging and TACC3 expression, were entered into the univariate Cox regression analysis in Table 2 to assess their impact on the OS of ESCC patients. The variables demonstrated to be significant in the univariate analysis were further analyzed by multivariate analysis. The multivariate analysis model revealed predominantly independent predictors of OS were TACC3 expression (HR, 1.515; 95% CI 1.053–2.180; *p* = 0.025), stage (HR, 1.54; 95% CI 1.071–2.214; *p* = 0.020) and alcohol intake (HR, 1.603; 95% CI 1.085–2.368; *p* = 0.018) presented in Table 2.

**Table 2:** Univariate analysis and multivariate analysis for predictors of overall survival

Variables	Univariate Analysis			Multivariate Analysis		
	HR	CI	*P* value	HR	CI	*P* value
Age	1.251	0.875–1.789	0.219	…	…	…
Gender	0.717	0.474–1.083	0.114	…	…	…
Smoke	1.241	0.853–1.804	0.259	…	…	…
Alcohol Intake	1.565	1.059–2.311	0.025*	1.603	1.085–2.368	0.018*
Grade	1.209	0.–1.949	0.425	…	…	…
Tumor Location	0.903	0.679–1.200	0.482	…	…	…
Surgery	1.035	0.860–1.247	0.713	…	…	…
TNM stage	1.577	1.099–2.263	0.013*	1.54	1.071–2.214	0.02*
TACC3	1.548	1.078–2.224	0.018*	1.515	1.053–2.180	0.025*

Variables: age, > 57 vs. ≤ 57; gender, female vs. male; smoke, smoker vs. non-smoker; alcohol consumption, yes vs no; grade, grade 3 vs. grade 2 vs. grade 1; tumor location, lower thoracic vs. middle thoracic vs. upper thoracic; surgery, left transthoracic approach vs. Ivor Lewis or McKeown; stage, III vs. II;. Cox proportional hazards model; … represent “date not available”;

* represent *P* < 0.05. HR, hazard ratio; CI, confidence interval.

### Knockdown of TACC3 suppresses the proliferation and clonogenicity of ESCC cells

**Figure 5:**
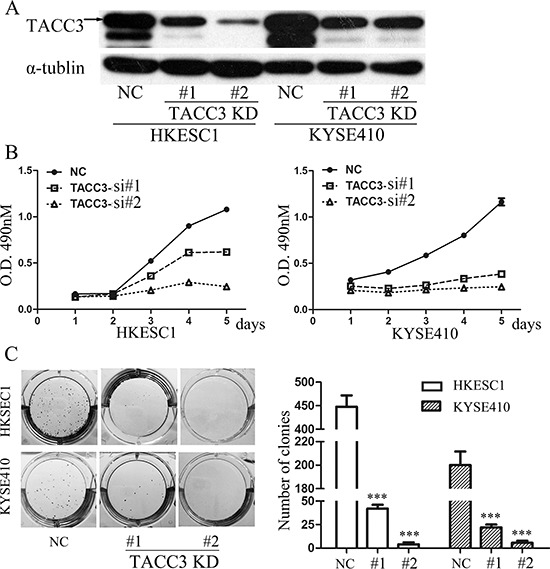
Knockdown of TACC3 suppresses the proliferation and clonogenicity of ESCC cells **(A)** Western blot lysates from HKESC1 and KYSE410 cells transfected with NS or TACC3-targeting (KD) siRNAs, with α.-tublin as an internal control. **(B)** Growth curves of HKESC1 and KYSE410 cells transfected with NS or TACC3-targeting (KD) siRNAs. Data represent means ± SD. **(C)** Soft agar colony formation assay of HKESC1 and KYSE410 cells infected with NS or TACC3-targeting (KD) shRNAs. The means ± SD in colony number compared to control NS cells across three independent experiments is shown (***p < 0.001).

To investigate the potential roles of TACC3 in ESCC tumorigenesis, we knocked down TACC3 in HKESC1 and KYSE410 cells with two siRNA duplexes. Downregulation of TACC3 was confirmed by Western blotting assay (Figure 5A). We next determined the cell viability by MTT assay at the indicated times. Compared to the negative control (NC), siTACC3 treatments caused markedly lower proliferation rate (Figure 5B). In addition, knock-down of TACC3 in HKESC1 and KYSE410 cells resulted in dramatically decreases both in the size and the number of colonies to grow in soft agar (Figure 5C). These results suggested the growth-promoting role of TACC3 in ESCC cells.

### TACC3 silencing inhibited ESCC cell migration

**Figure 6:**
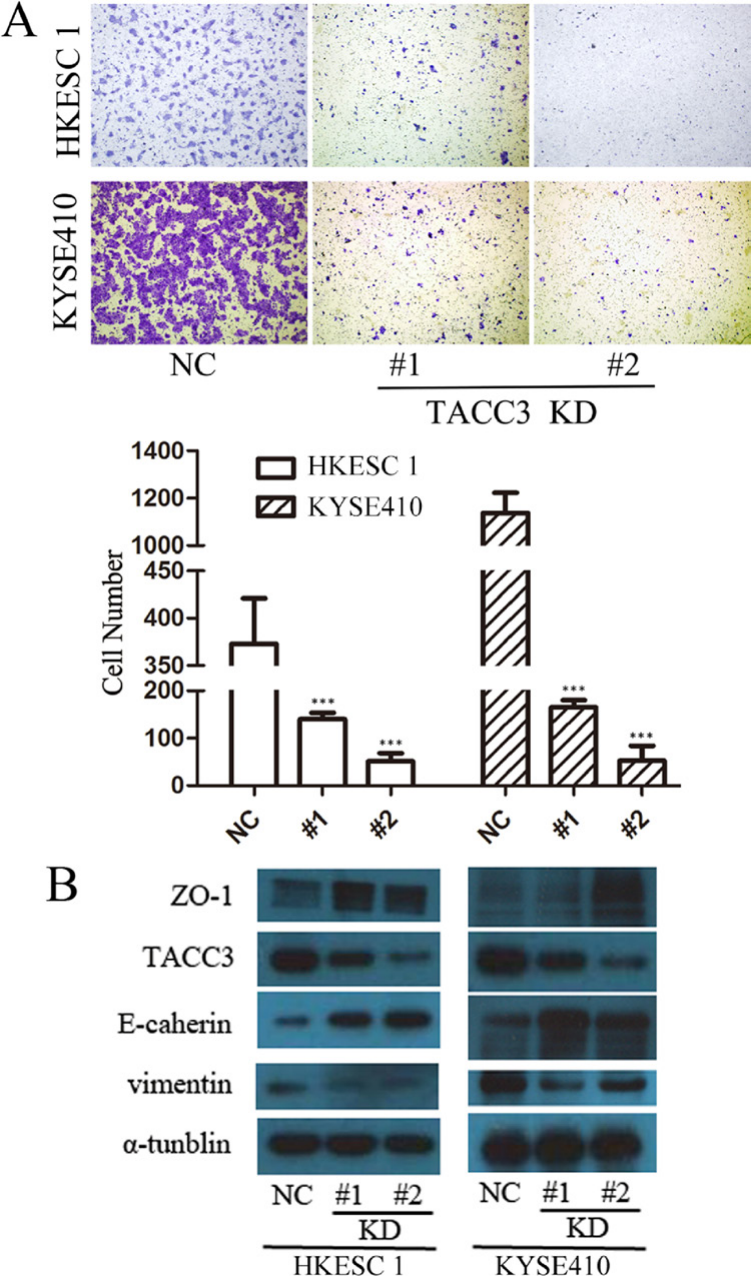
TACC3 silencing inhibited ESCC cell migration **(A)** Transwell assay of HKESC1 and KYSE410 cells transfected with NS or TACC3-targeting (KD) siRNAs. Data represent means ± SD. Data presented as means ± SD (***p < 0.001). **(B)** Western blot lysates from HKESC1 and KYSE410 cells transfected with NS or TACC3-targeting (KD) siRNAs, with α.-tublin as an internal control.

To verify the correlation between TACC3 and metastasis in ESCC cell lines, the migration ability of HKESC1 and KYSE410 cell, were compared by using transwell assays. After 12 hours incubation, the percentage of migrated cells post siTACC3 transfection was significantly less than the NC (Figure 6A). EMT is vital for morphogenesis during embryonic development and a key developmental program that is often activated during cancer invasion and metastasis [26]. Increasing observations of human tumors and experimental animal models have provided convincing evidence for its physiological relevance to tumorigenesis and cancer metastasis [27]. To study the mechanism by which TACC3 regulates cell migration, we examined the levels of EMT-associated protein in HKESC1 and KYSE410 cells post transfection. We found that suppressing TACC3 expression decreased the expression of vimentin, while elevated E-cadherin and ZO-1 expression (Figure 6B), indicating that TACC3 plays a role in EMT regulation of ESCC cells.

## DISCUSSION

Esophageal carcinoma is the sixth leading cause of cancer-related mortality and the eighth most common cancer worldwide. The past two decades have seen substantial progression in evidence relevant to various aspects of the treatment of esophageal carcinoma [28–30]. However, the overall 5-year survival rate still ranges from 15% to 25% due to poor recognize of mechanism and complexity of esophageal carcinoma genesis [31, 32]. Therefore, it is urgent to identify a marker to diagnose ESCC and predict prognosis of patients with ESCC.

It has been suspected that deregulation of TACC3 may be associated with the development of various types of human cancer [33, 34]. However, whether TACC3 acts as a tumor suppressor or an oncogene was controversial among studies [34]. Alternatively, TACC3 may have different functions depending on the type of cell, organ or carcinoma [18]. In this study, we aimed to investigate the biological significance of TACC3 in ESCC. We found that the expression of TACC3 protein is up-regulated in both ESCC cell lines and tissues. In addition, the expression level of TACC3 is positive correlated to the progression of ESCC. Moreover, the expression of TACC3 in ESCC patients with lymphoid nodal metastasis is significantly higher than that of ESCC patients without lymphoid nodal metastasis. To further define the clinical importance of TACC3, we investigated the correlation of TACC3 expression and overall survival of ESCC patients. We found that the high expression level of TACC3 protein in ESCC corresponds remarkably with patients’ poor survival time. Moreover, compared to the advanced stages, a stronger correlation between high TACC3 expression and short survivals was found at the early stages.

These results are accordant to the reports showing the oncogenic of TACC3 in multiple myeloma [9, 19], lung [20], bladder [21, 22], cervix uteri [18], breast [23] and ovarian cancer [35, 36]. Overall, in this study we first show that TACC3 might be an oncogene in ESCC and play a role in ESCC development, differentiation and lymph nodal metastasis.

Nowadays, neoadjuvant or adjuvant chemoradiotherapy plays a pivotal role in esophageal cancer patients with a locally advanced stage, and it has displayed a significantly survival benefit [30, 37]. Recently, scientists reported TACC3 was potential chemotherapy target. Schneider et al. showed that TACC3-depleted cells were highly susceptible to low-dose paclitaxel treatment, even in the presence of high levels of antiapoptotic p21 and active Akt [14]. Schmidt et al. suggested TACC3-depleted cells arrested in G (1) through a cellular senescence program and the onset of senescence following TACC3 knockdown was remarkably accelerated in the presence of paclitaxel concentrations [13]. Moreover, Yao et al. provided *in vivo* evidence that TACC3 loss led to tumor regression, accompanied by massive apoptosis in tumor tissue but none of overt abnormalities in normal tissues [38]. Additionally, it has been reported that high expression of TACC3 conferred cellular sensitization to radiation [39]. Growing evidence showed the presence of the fusion gene-FGFR3-TACC3, which might be an additional reason to cause the high expression of TACC3 [25, 40]. Moreover, Yao et al [41] identified a novel small compound called spindlactone (SPL) which can interact with and block the function of the TACC3-TOGp complex and finally induce spindle aberrations and tumor growth suppression. Interestingly, Ohoka et al [42] designed and synthesized novel small molecules called SNIPER(TACC3)s which target TACC3 and induce TACC3 poly-ubiquitylation and proteasomal degradation. These findings suggest that TACC3 is a potential anti-cancer molecular drug target. Based on our results, TACC3 was significantly higher in ESCC tissues than that in the normal esophageal tissues and was also higher in the patients with lymphoid metastasis than that in the patients without lymphoid metastasis. TACC3 may act as a significantly predictive biomarker for neoadjuvant or adjuvant chemoradiotherapy. However, patients with chemoradiotherapy were not enrolled in the current study. Therefore, a deeply investigation is needed for validating this hypothesis and elaborating the mechanisms.

EMT has been implicated in the progression to distant metastatic disease [27]. In a multifaceted genomic evaluation, TACC3 was established as a potential oncogene [39, 43]. In our study, TACC3 was found to be involved in proliferation and EMT of ESCC *in vitro*. The results that down-regulation of TACC3 in ESCC reduced the migration ability of ESCC cells suggested TACC3 might be a modulator in controlling metastasis of ESCC. Down-regulation of mesenchymal marker vimentin and up-regulation of epithelial markers E-cadherin and ZO-1 confirmed the role of TACC3 in EMT of ESCC. Its exact mechanisms, however, remain for a large scale of clinical investigation and further experimental exploration.

In summary, we observed the highly expressed TACC3 in ESCC and knockdown TACC3 contributed decreases in cell proliferation, colony formation ability and EMT of ESCC cells, and demonstrated that ESCC patients expressing high levels of TACC3 exhibit a substantially lower 5-year overall survival rate than TACC3-low expression patients. A high expression of TACC3 in ESCC patients is positively associated with high histological grade and lymphoid metastasizing status. Taken together, this novel study identifies TACC3 not only as a useful biomarker to diagnose and determine the prognosis of ESCC, but also as a potential therapeutic target for patients with ESCC.

## MATERIALS AND METHODS

### Patients and tissue specimens

Twenty eight ESCC biopsy samples and their adjacent paired noncancerous esophageal tissues used for qRT-PCR and Western blotting were collected from Sun Yat-sen University Cancer Center (SYSUCC), Guangzhou, China. These tissues were frozen in liquid nitrogen and stored during 2013. For IHC analysis, 209 paraffin-embedded ESCC specimens and 164 matched carcinoma-adjacent tissues were collected in SYSUCC from 2001 to 2007. All the ESCC patients were histologically and clinically diagnosed and were treated with radical surgery and without neoadjuvant/adjuvant treatments. Follow-up information was available for all these patients. Pathological stage was recorded according to the 7th edition of the Union for International Cancer Control-TNM (UICC-TNM) Classification [44]. This study was approved by the committee for ethical review of research involving human subjects at SYSUCC. The clinical characteristics of the ESCC patients were described in Table 1.

### Human esophageal cell lines

The normal human esophageal cell line NE3 and the ESCC cell line Eca-109 were obtained from Dr. Jin (the University of Hong Kong, P. R. China) [45]. The ESCC cell lines EC18, HKESC1, KYSE30, KYSE140, KYSE150, KYSE410, and KYSE510 were kindly provided by Professor Xin-Yuan Guan (Department of Clinical Oncology, The University of Hong Kong) [46]. NE3 was cultured in Keratinocyte-SFM media (Invitrogen, Grand Island, USA) with the addition of growth supplements provided by the manufacturers (the exact content of the growth supplements was not stated by the manufacturers). All tumor cell lines were cultured in DMEM (Invitrogen) supplemented with 10% fetal bovine serum (FBS; Hyclone, Logan, UT), and in a humidified 5% CO2 incubator at 37°C.

### SiRNA transfection

Two siRNAs targeting the TACC3 [GenBank: NM_006342.2] were denoted as siTACC3-#1 (GCATGCACGGTGCAAATGA) and siTACC3-#2 (CCACAGATCTGAACTCCAT). The negative control (NC) was indicated as siNC which was nonhomologous to any human genome sequences. The HKESC1 and KYSE410 cells were transfected with 40 nM of the RNA duplex and 4 μL of Lipofectamine RNAiMAX (Invitrogen), according to the manufacturer's instructions. Cells were culture for 48 h and harvested for further experiments.

### Quantitative RT-PCR analysis

Total RNA was isolated from NE3, ESCC cell lines and tissue specimens by using the TRIzol reagent (Invitrogen) according to the manufacturer's instructions. RNA concentration and quanlity were determined with NanoDrop spectrophotometer (ND-1000, Thermo Scientific, Massachusetts, USA). Complementary DNA (cDNA) was synthesized using 2 μg of the total RNA according to a reverse transcriptase kit (Invitrogen). qRT-PCR with the Power SYBR Green qPCR SuperMix-UDG (Invitrogen) was used to measure the mRNA level of the target genes on an ABIPrism-7500 Sequence Detector System (ABI, Applied Biosystems, Carlsbad, USA). β-actin was used as an internal control. Relative expression of the TACC3 was normalized to the expression of β-actin, which yielded a 2^−△Ct^ value. The primer sequences were as follows: TACC3 sense 5′-CCTCTTCAAGCGTTTTGAGAAAC-3′, TACC3 antisense 5′-GCCCTCCTGGGTGATCCTT-3′, β-actin sense 5′-CGCGAGAAGATGACCCAGAT-3′, β-actin antisense 5′-GGGCATACCCCTCGTAGATG-3′. All reactions were run in triplicate.

### Western blotting analysis

Equal amounts of whole tissue or cell lysates were electrophoretically separated in a 9% SDS polyacrylamide gel electrophoresis (PAGE) and transferred to a polyvinylidene difluoride (PVDF) membrane (Pall, Port Washington, USA). 5% skimmed milk was used to block the membrane for 1 hour (h) at room temperature. Then the membrane was incubated with the indicated antibodies overnight at 4°C. The signals were detected using an enhanced chemiluminescence (GE Healthcare, New Jersey, USA). The membranes were stripped and probed with a mouse monoclonal antibody against human α-tubulin (diluted 1:3000; Santa Cruz Biotechnology, Texas, USA) to confirm equal loading of the samples.

### Immunohistochemical analysis

The paraffin-embedded ESCC samples were cut into 4-μm sections. Then the sections were baked for 1 hour at 60°C, deparaffinized in xylenes and rehydrated with alcohol to distilled water. Three percent hydrogen peroxide was used to block endogenous peroxidase activity at room temperature for 10 min, and then the sections were boiled in Citrate Antigen Retrieval Solution (pH = 6.0) for 5 min in a electric pressure cooker antigen retrieval. After that, the sections were incubated with the rabbit polyclonal anti-TACC3 antibody (diluted 1:800, Abcam, USA) overnight at 4°C in a moist chamber and a secondary antibody for 30 min at 37°C on the next day. Subsequently, the sections were stained for protein detection in 3, 3-diaminobenzidine for 2 min and counterstained with Mayer's hematoxylin to stain nucleus. Finally, the sections were dehydrated and mounted. As a negative control, the primary antibody was replaced by normal rabbit serum.

Each section was independently evaluated by two pathologists who were blinded to the clinical status of the patients. The biomarker was evaluated according to staining intensity scored as (0, negative staining; 1, weak staining; 2, moderate staining; 3, strong staining) and the extent of staining scored as the percentage of positive cells. The final immunoreactivity score (IRS) is the product of intensity score and the extent score.

### Proliferation assay *in vitro*

MTT (methyl thiazolyl tetrazolium, Sigma-Aldrich, Missouri, USA) assay was used to measure cell proliferation in ESCC cells. Briefly, after transfection with siNC, siTACC3-#1 or siTACC3-#2 for 48 h, ESCC cells (2500 per well) were plated in 96-well plates with RPMI1640 containing 10% FBS. Each sample had six replicates. At the indicated time points, cells were added with 10 μL of 5 mg/mL MTT tetrazolium and incubated for 4 h at 37°C. To dissolve the precipitates, 200 μL of DMSO was added to each well for 20 min after discarding the media. The absorbance of the solution was read at 490 nm using the Spectramax M5 (Molecular Devices, Sunnyvale, USA).

### Colony formation assay

Cells (200 cells per well) were plated evenly in 6-well plates and cultured for 14 days. After fixed with methanol for 10 min, the colonies were stained with 0.5% crystal violet in 20% methanol and counted. Independent triplicate experiments were performed.

### Transwell assay

HKESC1 or KYSE410 (8 × 10^4^ cells) with 200 μL FBS-free RPMI were seeded in the top chamber of the transwell (BD Biosciences, San Jose, USA) at 48 h post-transfection. The lower chamber was filled with DMEM with 10% FBS as a chemo-attractant. After incubation for 24 hours, cells on the lower surface of the member were fixed, stained and counted. Each group of cells was done in triplicate.

### Statistical analysis

Data were analyzed by the SPSS standard version 17.0 (SPSS, Chicago, USA). The Kaplan–Meier method was used to estimate overall survival (OS) and multivariate analysis was performed by the Cox proportional hazards model. The chi-square test was used to analyze the relationship between TACC3 expression and the clinicopathological characteristics. Analysis of the differences between groups was determined with the two-tailed Mann–Whitney test. *P* values less than 0.05 were considered statistically significant.

## SUPPLEMENTARY TABLE


